# Persistent sex disparities in access to dolutegravir‐based antiretroviral therapy in Latin America and the Caribbean: results from a retrospective observational study using data from 2017 to 2022

**DOI:** 10.1002/jia2.26470

**Published:** 2025-07-09

**Authors:** Fernanda F. Fonseca, Paridhi Ranadive, Bryan E. Shepherd, Flavia G. F. Ferreira, Maria F. Rodríguez, Daisy M. Machado, Vanessa Rouzier, Diana Varela, Fernanda Maruri, Peter Ribeiro, Beatriz Grinsztejn, Sandra Wagner Cardoso, Valdiléa G. Veloso, Jessica L. Castilho, Emilia M. Jalil, CCASAnet

**Affiliations:** ^1^ AIDS Health Care Foundation, Global Program Sao Paulo Brazil; ^2^ Fiocruz, Instituto Nacional de Infectologia Evandro Chagas Rio de Janeiro Brazil; ^3^ Department of Biostatistics Vanderbilt University Medical Center Nashville Tennessee USA; ^4^ Departamento de Pediatria UFMG, Faculdade de Medicina Belo Horizonte Brazil; ^5^ Hospital Clínico San Borja Arriarán Fundación Arriarán Santiago Chile; ^6^ UNIFESP, Escola Paulista de Medicina Disciplina de Infectologia Pediátrica Sao Paulo Brazil; ^7^ Groupe Haitien d'Etudes du Sarcome de Kaposi et des Infections Opportunistes Port‐au‐Prince Haiti; ^8^ Instituto Hondureño de Seguridad Social & Hospital Escuela Universitario Tegucigalpa Honduras; ^9^ Division of Infectious Diseases Department of Medicine, Vanderbilt University Medical Center Nashville Tennessee USA

**Keywords:** disparities, dolutegravir, gender, HIV, sex, women

## Abstract

**Introduction:**

Despite its reversal in July 2019, the World Health Organization warning issued in May 2018 of potential teratogenicity associated with dolutegravir (DTG) may have produced persistent sex disparities in access to DTG. We compared DTG uptake of people with HIV (PWH) by sex in Latin America and the Caribbean (LAC) and its potential impact on virologic outcomes.

**Methods:**

We evaluated DTG initiation among antiretroviral therapy (ART)‐naïve and ‐experienced cisgender PWH ≥16 years of age after DTG availability in Brazil (February/2017), Chile (August/2019), Haiti (November/2018) and Honduras (December/2018). Time was divided into pre‐ (before May/2018), during‐ (May/2018−July/2019) and post‐ (after July/2019) warning periods. We examined interactions of sex, age and calendar era with multivariable modified Poisson regression models and Cox proportional hazard models for the outcomes of DTG initiation among ART‐naïve and ART‐experienced PWH, respectively, and HIV RNA <50 copies/ml in the first year of therapy among ART‐naïve PWH, adjusting for site and tuberculosis.

**Results:**

Among 4622 ART‐naïve PWH, 3853 (83%) initiated DTG. ART‐naïve females aged 16–49 years were less likely to initiate DTG compared to males of the same age both in the pre/during‐warning (adjusted prevalence ratio [aPR]: 0.75 [95% confidence interval (95% CI): 0.71−0.80]) and in the post‐warning periods (aPR: 0.97 [95% CI: 0.95−1.00]). Among 16,154 ART‐experienced PWH, 9236 (57%) initiated DTG. ART‐experienced females 16–49 years were less likely to initiate DTG compared to males of the same age in the pre/during‐warning (adjusted hazard ratio [aHR]: 0.69 [95% CI: 0.66−0.73]) and post‐warning periods (aHR: 0.79 [95% CI: 0.70−0.90]). This sex difference was not observed among older ART‐experienced females and males pre/during‐warning (aHR: 1.06 [95% CI: 0.99−1.14]). Compared to starting ART without DTG, DTG‐based ART use was associated with a higher likelihood of HIV RNA suppression in the first year (aPR = 1.10 [95% CI: 1.04−1.16]). In the post‐warning period, females aged 16–49 years had a likelihood of viral suppression similar to males of the same age (aPR: 1.03 [95% CI: 0.96−1.10]), which did not change after adjusting for DTG use (aPR: 1.03 [95% CI: 0.97−1.11]).

**Conclusions:**

Despite the updated guidelines recommending DTG for all PWH, there are persistent sex disparities in the access to DTG in LAC, especially among females within the reproductive age.

## 1 INTRODUCTION

Dolutegravir (DTG) is a highly potent second‐generation integrase strand transfer inhibitor (INSTI) antiretroviral therapy (ART) with demonstrated good efficacy and tolerability [1, 2]. Due to its several advantages, DTG is preferred over previous ART by the World Health Organization (WHO) and in other international and national HIV treatment protocols [3, 4, 5, 6, 7, 8, 9]. In May 2018, based on preliminary results of the Tsepamo study in Botswana, the WHO issued a warning on a potential increased risk of neural tube defects (NTDs) in infants born to women on DTG by the time of conception [10, 11, 12, 13]. Based on this warning, the use of DTG by women was questioned and even limited in several countries [14, 15].

Updated results from the Tsepamo study and others showed no association between NTD and DTG [16, 17]. Since July 2019, WHO has recommended DTG for first‐, second‐ and third‐line ART and reinforced the superiority of DTG‐based ART for both cisgender men and women, including those with reproductive potential and those who are pregnant [6]. In 2021, results of a multicentre, open‐label, randomized controlled, phase 3 trial showed that ART containing DTG initiated during pregnancy had superior virological efficacy at delivery compared with the efavirenz‐based ART and the lowest frequency of composite adverse pregnancy outcomes [18].

Despite these reassurances, concerns about DTG safety around conception and pregnancy may have impacted the clinical use of DTG among females with HIV of reproductive age. The WHO's DTG warning came during the transition to DTG‐based ART first‐line regimens in many low‐ and middle‐income countries [19, 20]. We sought to assess potential lasting sex and age disparities in DTG initiation among females and males with HIV in Latin America and the Caribbean (LAC) before, during and after WHO warning of a potential teratogenic effect. We also sought to examine if those differences resulted in sex disparities in virologic outcomes.

## 2 METHODS

### 2.1 Study population and design

We included retrospective, observational data of adults (aged ≥16 years) with HIV receiving ART in sites with wide DTG availability from the Caribbean, Central and South America network for HIV epidemiology (CCASAnet) clinical sites in Brazil (Instituto Nacional de Infectologia Evandro Chagas/Fiocruz, Rio de Janeiro; Universidade Federal de Sao Paulo, Sao Paulo; Universidade Federal de Minas Gerais, Belo Horizonte), Chile (Fundacion Arriaran, Santiago), Haiti (Les Centres GHESKIO, Port‐au‐Prince) and Honduras (Hospital Escuela and Instituto Hondureno de Seguridad Social, Tegucigalpa). Other CCASAnet sites in Argentina, Mexico and Peru were not included due to limited DTG use during the study period. CCASAnet sites collect and regularly send data to the data coordinating centre at Vanderbilt University, US, for data harmonization, quality checks and processing [21]. Ethics committees at all sites and Vanderbilt approved the project, waiving the requirement of individual informed consent.

We included all females and males with HIV (PWH) who entered or were receiving clinical care after the time that DTG became available for general use as first‐line ART in each of the clinical sites included in the analysis (Brazil: February 2017 for ART‐naïve PWH and January 2018 for ART‐experienced PWH; Chile: August 2019; Haiti: November 2018; and Honduras: December 2018). We excluded individuals: (1) exposed to DTG prior to clinic entry or prior to general availability for first‐line ART; (2) whose ART exposure before clinic entry was unknown; (3) who started ART at <16 years of age; (4) who did not receive any ART after the date of DTG availability; and (5) who were identified as transgender due to their limited number (*n* = 64) and our primary focus on sex disparities. Each site contributed longitudinal data until the 95th percentile of the last observation date (December 2021 in Brazil, October 2022 in Chile, January 2022 in Honduras and March 2022 in Haiti); these dates were considered as each site's cohort closure date.

To account for potential differences in DTG use related to previous ART, we analysed DTG initiation according to prior ART use (i.e. PWH who were ART‐naïve and first started ART after DTG availability and PWH who were ART‐experienced at the time of DTG availability). For ART‐naïve individuals, baseline was time at ART initiation. For ART‐experienced individuals, baseline was the first clinical activity after general DTG availability in their clinical site. For the outcome of DTG initiation, we used cross‐sectional analyses among ART‐naïve individuals, and we used time‐to‐event analyses among ART‐experienced individuals. In time‐to‐event analyses, censorship occurred at the first occurrence of DTG‐initiation, last clinic visit or death.

### 2.2 Outcome and exposure definitions

Our primary outcome was DTG initiation. We also assessed the secondary outcome of virologic suppression (defined as HIV RNA <50 copies/ml) at any time within 12 months of ART initiation among ART‐naïve individuals with at least 12 months of follow‐up after ART initiation.

We examined sex as a binary variable (cisgender males and females). We divided time into periods relative to the WHO warning: pre‐ (before May 2018), during (May 2018−July 2019) and post‐warning periods (after July 2019). We categorized age into two groups: 16–49 and ≥50 years. We used a three‐way interaction term combining sex, period and age to dynamically estimate disparities of DTG initiation across these parameters.

Other variables included demographics, clinical site, CD4 cell count (collected within 180 days before to 30 days after baseline), HIV RNA (collected within 180 days before to 7 days after baseline), and prevalent tuberculosis and other opportunistic infections (diagnosed within ± 30 days of baseline). For ART‐experienced individuals, we gathered information on current ART by drug class and number of prior ART regimens at baseline.

### 2.3 Statistical approach

We compared sex differences in baseline characteristics of ART‐naïve and ART‐experienced cohorts using Pearson chi‐square or Wilcoxon rank‐sum tests, as appropriate. We examined the crude proportion of ART‐naïve and ART‐experienced males and females who initiated DTG by calendar year across each country of site. Denominators included all ART‐naïve PWH initiating ART and ART‐experienced PWH in care within a 6‐month window eligible to initiate DTG; numerators included ART‐naïve PWH who started DTG and ART‐experienced who switched to DTG within the 6‐month window.

We graphically examined the predicted probability of DTG initiation among ART‐naïve and ART‐experienced males and females separately using modified Poisson regression models that included continuous measures of time and baseline age using natural splines with three knots to relax assumptions of linearity, adjusting for presence of tuberculosis and study site, and then weighted by the size of the study site to obtain cross‐site estimates.

We used modified Poisson regression models to calculate adjusted prevalence ratios (aPR) for DTG initiation among ART‐naïve individuals which included main effects, two‐ and three‐way interaction terms for sex, period and age, including period and age as categorical variables, and adjusting for site and presence of tuberculosis. We used multivariable Cox proportional hazard models to examine the likelihood of DTG initiation among ART‐experienced PWH, similarly including the sex‐period‐age interaction terms, adjusting for time‐updated tuberculosis, number of prior ART regimens at baseline and time‐updated HIV RNA >1000 copies/ml. All Cox models included stratification by site in the analyses to allow for differing underlying rates of DTG use across sites. Multiple imputations with 20 replications were used for missing HIV RNA. Given differences in cohort composition by sex and HIV treatment policies, analyses were repeated and stratified by Haiti and all other sites in sensitivity analyses.

Lastly, to examine whether sex differences in DTG use contributed to HIV treatment outcomes, we examined the likelihood of achieving HIV RNA <50 copies/ml in the first year following ART initiation among ART‐naïve PWH. Including all ART‐naïve PWH with at least 1 year of follow‐up and ≥1 HIV RNA value in the first year, we used multivariable modified Poisson regression models to examine the association of virologic suppression by sex‐period‐age groups with and without including adjusting for DTG use, adjusting for site and tuberculosis at ART initiation. Multivariable models were also repeated, stratified by Haiti and all other sites. In a sensitivity analysis, individuals who were excluded for missing HIV RNA were included as virologic failure outcomes.

All analyses were conducted in R version 3.4.3. The analysis code is posted at https://biostat.app.vumc.org/ArchivedAnalyses.

## 3 RESULTS

**Figure 1:**
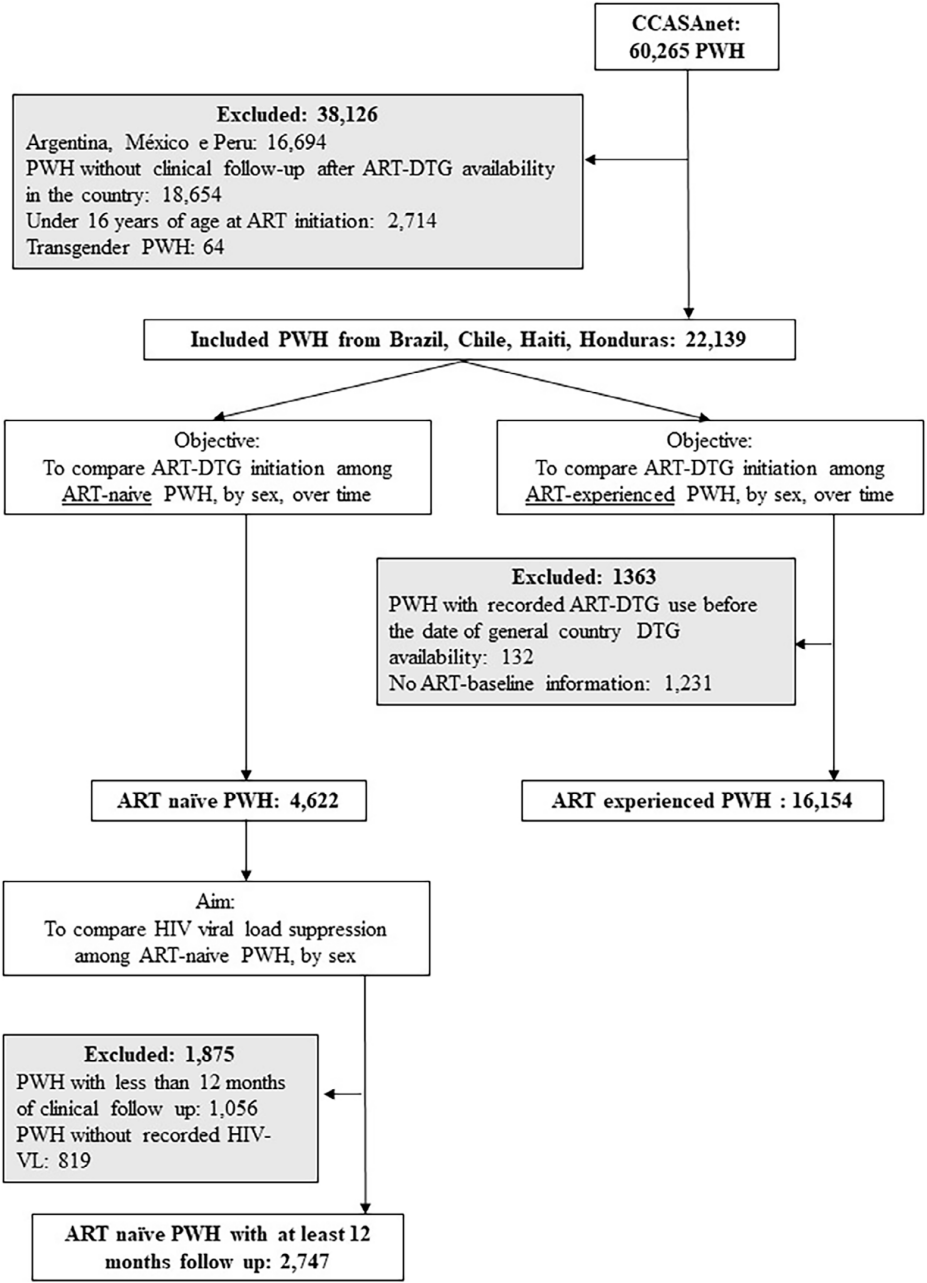
Flow diagram of cohort inclusion/exclusion criteria. Abbreviations: ART‐DTG, antiretroviral therapy containing dolutegravir; CCASAnet, Caribbean, Central and South America network for HIV epidemiology; PWH, people with HIV; VL, viral load.

We identified 22,139 eligible PWH, of which 4622 were ART‐naïve and 16,154 were ART‐experienced (Figure 1). Overall, the majority of PWH included were aged between 16 and 49 years and the majority of PWH were from the clinical site in Haiti (Table 1). While 38% of PWH included in the ART‐naïve group were female, the ART‐experienced group was more balanced for sex. ART initiation among ART‐naïve PWH most frequently occurred in the post‐warning period, while baseline study entry occurred most frequently during the WHO warning period among ART‐experienced PWH. DTG became widely available in the pre‐warning period only in Brazil, in Haiti during the warning period and only post‐warning in Honduras and Chile (Figure S1). In unadjusted estimates, a higher proportion of ART‐naïve males than ART‐naïve females started DTG‐containing ART in clinical sites in Brazil and Haiti during the beginning of the DTG rollout in those countries (Figure S1).

**Table 1:** Characteristics of treatment naïve and experienced PWH at baseline^a^, overall and stratified by sex, from 2017 to 2022

	ART‐naïve PWH				ART‐experienced PWH			
	All N = 4622	Females N = 1762	Males N = 2860	p‐value^b^	All N = 16,154	Females N = 8051	Males N = 8103	p‐value^b^
**Age in years, median (IQR)**	34 (27−42)	35 (28−44)	33 (27−41)	<0.001	36 (29−45)	36 (29−44)	37 (30−45)	<0.001
**Age group, n (%)**								0.012
16−49 years	4055 (88)	1519 (86)	2536 (89)		14,044 (87)	7053 (88)	6991 (86)	
≥ 50 years	567 (12)	243 (14)	324 (11)		2110 (13)	998 (12)	1112 (14)	
**Country site, n (%)**				<0.001				<0.001
Brazil	1193 (26)	160 (9)	1033 (36)		3216 (20)	960 (12)	2256 (28)	
Chile	751 (16)	92 (5)	659 (23)		914 (6)	81 (1)	831 (10)	
Haiti	2605 (56)	1496 (85)	1109 (39)		11,582 (72)	6783 (84)	4799 (59)	
Honduras	73 (2)	14 (1)	59 (2)		442 (3)	225 (3)	217 (3)	
**Time period, n (%)**				<0.001				<0.001
Pre‐warning	315 (7)	52 (3)	263 (9)		3151 (20)	942 (12)	2209 (27)	
During warning	1195 (26)	584 (33)	611 (21)		10,901 (68)	6330 (79)	4571 (56)	
Post‐warning	3112 (67)	1126 (64)	1986 (69)		2102 (13)	779 (10)	1323 (16)	
**HIV RNA (log_10_), median (IQR)**	4.7 (4.0−5.3)	4.4 (3.8−5.0)	4.7 (4.1−5.3)	<0.001	0.0 (0.0−3.1)	0.0 (0.0−3.5)	0.0 (0.0−1.9)	<0.001
Missing	2791	1426	1365		3021	1467	1554	
**CD4 cell count (cells/mm^3^), median (IQR)**	325 (151−539)	339 (154−556)	320 (150−530)	0.12	572 (353−792)	603 (347−852)	561 (356−768)	0.013
Missing	1689	909	780		13,464	7320	6144	
**TB history, n (%)**	157 (3)	64 (4)	93 (3)	0.5	135 (1)	49 (1)	86 (1)	0.002
**AIDS‐defining illness (except TB), n (%)**	303 (7)	59 (3)	244 (9)	<0.001	53 (<1)	14 (<1)	39 (1)	<0.001
**ART regimen, n (%)**				<0.001				<0.001
DTG‐based	3853 (83)	1498 (85)	2355 (82)		NA	NA	NA	
INSTI‐other based^c^	345 (8)	41 (2)	304 (11)		377 (2)	49 (1)	328 (4)	
NNRTI‐based	298 (7)	164 (9)	134 (5)		14,189 (88)	7215 (90)	6974 (86)	
PI‐based	113 (2)	58 (3)	55 (2)		358 (2)	611 (8)	619 (8)	
Other^d^	12 (<1)	1 (<1)	12 (1)		1230 (7)	176 (1)	182 (2)	
**Switch to DTG, n (%)**					9236 (57)	5019 (62)	4217 (52)	<0.001
**Number of previous ART regimens, n** **(%)**								0.7
1					11,622 (72)	5780 (72)	5842 (72)	
>1					4532 (28)	2261(28)	2271 (28)	
**Follow‐up time (years) median (IQR)**					0.5 (0.0−2.5)	0.4 (0.0−2.3)	0.7 (0.0−2.8)	<0.001
**Death, n (%)**					697 (4)	314 (4)	383 (5)	0.01

Abbreviations: ART, antiretroviral therapy; DTG, dolutegravir; INSTI, integrase inhibitor; IQR, interquartile range; NA, not applicable; NNRTI, non‐nucleotide reverse transcriptase inhibitor; PI, protease inhibitor; PWH, person living with HIV; TB, tuberculosis; VL, viral load.

^a^ Baseline time point refers to ART initiation among ART‐naïve PHIV and refers to earliest clinic time point after which DTG became available at each site for ART‐experienced PWH.

^b^ Wilcoxon rank sum test; Pearson's Chi‐squared test for comparisons of males and females for continuous and categorical measures, respectively.

^c^ INSTI‐other: not DTG based, for example raltegravir or bictegravir.

^d^ Other ART: fusion inhibitors (enfuvirtide) and R5 receptor antagonists (maraviroc).

**Figure 2:**
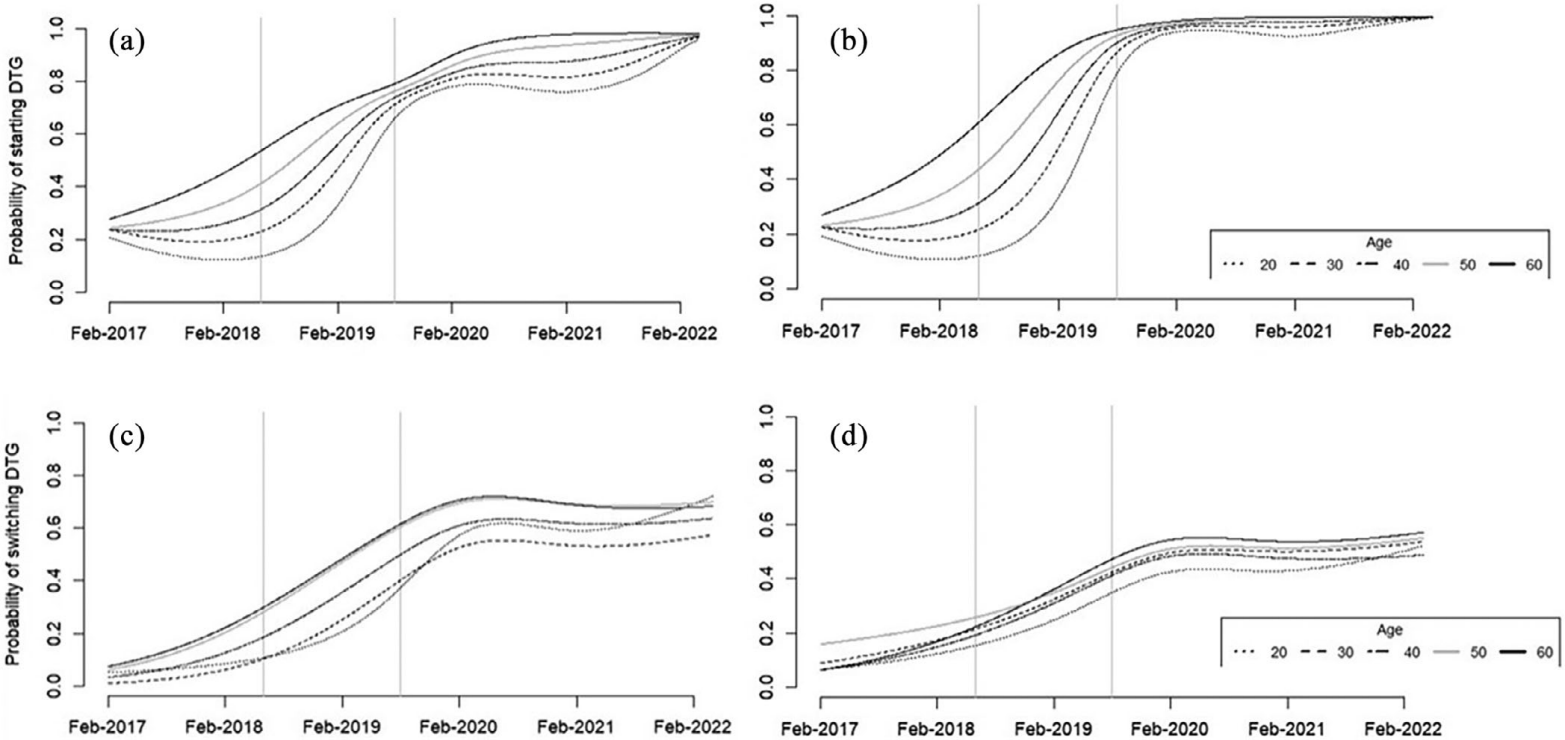
Weighted predicted probability of dolutegravir (DTG) initiation over time among ART‐naïve and ART‐experienced people with HIV by age and sex. (a) ART‐naïve females, (b) ART‐naïve males, (c) ART‐experienced females and (d) ART‐experienced males. Predicted probability of dolutegravir initiation was calculated using modified Poisson regression models that included continuous measures of time and baseline age using natural splines with three knots to relax assumptions of linearity and with weighted probabilities for sites, adjusting for the presence of tuberculosis and other AIDS‐defining illnesses. Vertical grey lines indicate the start (May 2018) and end (July 2019) of the warning period.

In Figure 2, among both ART‐naive (Figure 2a,b) and ART‐experienced (Figure 2c,d) PWH, younger females were less likely to start DTG compared to older females in the pre‐ and during‐warning periods. In post‐warning periods for both ART‐naive and ART‐experienced PWH, the difference in likelihood of DTG initiation by age narrowed among females but persisted. In contrast, among ART‐naïve PWH, younger males were more likely to initiate DTG than older males in the pre‐ and during‐warning periods, but the age differences resolved in the post‐warning period for males. Among ART‐experienced males, older males were slightly more likely to switch to DTG than younger males.

In multivariable models, among ART‐naive PWH, females aged 16–49 years were less likely to initiate DTG in both pre/during‐ and post‐warning periods compared to males of the same age, though the sex disparity decreased in the post‐warning period (Table 2). In contrast, there was no difference in DTG initiation by sex among adults ≥50 years of age in either the pre/during‐ or post‐warning periods. By age, ART‐naïve females aged 16–49 years were also less likely to initiate DTG compared to those males aged ≥50 years in both pre/during‐ and post‐warning periods. This difference by age was not observed among males in either period.

**Table 2:** Adjusted prevalence risk ratios and hazard ratios of starting dolutegravir among ART‐naïve and ‐experienced people with HIV by sex, period and age

Comparison	ART‐naïve PWH		ART‐experienced PWH	
	aPR (95%CI)^a^	*p*‐value	aHR (95%CI)^b^	*p*‐value
**Sex**				
16−49 years pre/during‐warning: females versus males	0.75 (0.71−0.80)	<0.001	0.69 (0.66−0.73)	<0.001
≥50 years pre/during warning: females versus males	1.03 (0.91−1.16)	0.64	1.06 (0.99−1.14)	0.09
16−49 years post‐warning: females versus males	0.97 (0.95−1.00)	0.03	0.79 (0.70−0.90)	<0.001
≥50 years post‐warning: females versus males	1.01 (0.96−1.06)	0.66	1.14 (0.95−1.38)	0.15
**Period**				
Females 16–49 years: pre/during versus post‐warning	0.69 (0.65−0.73)	<0.001	1.04 (0.94−1.15)	0.44
Males 16–49 years: pre/during versus post‐warning	0.89 (0.86−0.91)	<0.001	1.19 (1.05−1.36)	0.007
Females ≥50 years: pre/during versus post‐warning	0.83 (0.76−0.90)	<0.001	1.14 (0.95−1.32)	0.08
Males ≥50 years: pre/during versus post‐warning	0.82 (0.74−0.90)	<0.001	1.23 (1.06−1.42)	0.005
**Age**				
Females pre/during‐warning: 16–49 versus ≥50 years	0.79 (0.71−0.87)	<0.001	0.59 (0.56−0.63)	<0.001
Females post‐warning: 16–49 versus ≥50 years	0.95 (0.92−0.98)	<0.001	0.65 (0.55−0.75)	<0.001
Males pre/during‐warning: 16–49 versus ≥50 years	1.08 (0.98−1.18)	0.12	0.90 (0.85−0.96)	0.002
Males post‐warning: 16–49 versus ≥50 years	0.99 (0.97−1.06)	0.63	0.93 (0.92−1.26)	0.37

Abbreviations: 95% CI, 95% confidence interval; aHR, adjusted hazard ratio; aPR, adjusted prevalence risk ratio; ART, antiretroviral therapy; CI, confidence interval; DTG, dolutegravir.

^a^ Multivariable modified Poisson regression model including sex‐period‐age interaction term and additional covariates of site and tuberculosis at baseline.

^b^ Multivariable Cox proportional hazard model, including sex‐period‐age interaction term and additional covariates of tuberculosis at baseline, number of previous ART regimens, HIV RNA at baseline and stratified by site. Missing variables were multiply imputed.

Among ART‐experienced PWH, females aged 16–49 years were less likely to start DTG compared to similarly aged males in the pre‐/during‐warning periods (Table 2). The difference by sex for younger ART‐experienced PWH persisted and was similar in the post‐warning period. Again, there were no differences in sex by DTG initiation among older ART‐experienced PWH in either period. Similarly, differences in DTG initiation between younger and older females who were ART‐experienced were observed in the pre‐/during‐warning periods and persisted in the post‐warning period. While younger ART‐experienced males were less likely to start DTG in the pre‐/during‐warning period, there was no difference in DTG initiation by age for males in the post‐warning period.

Multivariable models also demonstrated that site was a strong predictor of starting DTG. Among ART‐naïve, PWH in Brazil (adjusted prevalence risk ratio [aPR]: 0.95 [95% confidence interval [CI] 0.92−0.97]), Chile (aPR: 0.49 [95% CI 0.45−0.53]) and Honduras (aPR: 0.46 [95% CI 0.36−0.58]) were less likely to initiate DTG compared with PWH in Haiti. Active tuberculosis at baseline was also associated with a decreased likelihood of initiating DTG both among ART‐naïve PWH (aPR: 0.72 [95% CI 0.65−0.80]) and ART‐experienced PWH (aHR: 0.78 [95% CI 0.64−0.96]). Multivariable sensitivity models for ART‐naïve and ART‐experienced PWH stratified by clinical sites in Haiti and all other countries were similar overall (Tables S1 and S2). DTG use among ART‐experienced PWH in clinical sites in Haiti compared to other countries showed different patterns of use among males (Table S2) and by individuals with time‐updated HIV RNA and >1 previous ART regimens.

From a total 2747 ART‐naïve PWH with an available HIV RNA measurement within 12 months of ART initiation, 2443 (89%) achieved HIV RNA <50 copies/ml (Table S3). Among the 2282 PWH who initiated DTG, 2068 (91%) achieved viral suppression. Among 190 and 212 PWH who initiated another INSTI or an NNTRI‐based regimen, 158 (83%) and 167 (79%) achieved viral suppression, respectively. In multivariable models, only DTG use was statistically associated with an increased likelihood of achieving HIV RNA <50 copies/ml in the first year (aPR: 1.10 [95% CI: 1.04−1.16]) (Table 3). In the post‐warning period, females aged 16–49 years had a likelihood of viral suppression similar to males of the same age (aPR: 1.03 [95% CI: 0.96−1.10]), which did not change after adjusting for DTG use (aPR: 1.03 [95% CI: 0.97−1.11]). There was no difference in viral suppression by sex or age or according to the warning period (Table S4). Results were similar when PWH with missing HIV RNA results in the first 12 months were included as not suppressed (Table S5).

**Table 3:** Adjusted prevalence risk ratio of achieving HIV RNA <50 copies/ml within first year of ART among ART‐naive people with HIV

	aPR (95% CI)^a^	*p*‐value
DTG versus no DTG	1.10 (1.04−1.16)	0.001
**Sex**		
16−49 years post‐warning: females versus Males	1.04 (0.97−1.11)	0.30
**Period**		
Males 16–49 years: pre/during versus post‐warning	1.02 (0.95−1.09)	0.59
**Age**		
Males post‐warning: ≥50 years versus 16–49 years	0.99 (0.95−1.04)	0.80

Abbreviations: 95% CI, 95% confidence interval; aPR, adjusted prevalence risk ratio; ART, antiretroviral therapy; CI, confidence interval; DTG, dolutegravir.

^a^ Multivariable modified Poisson regression model including sex‐period‐age interaction term and additional covariates of site and tuberculosis at baseline.

## 4 DISCUSSION

In this multinational observational cohort of PWH in LAC from 2017 to 2022, females aged 16−49 years were less likely to initiate DTG when compared to males in the same age group. This disparity was most significant among ART‐experienced PWH. Further, the sex disparity in DTG use among younger PWH persisted after the updates to the WHO recommendations in July 2019 and despite the accumulation of scientific evidence of the superiority and safety of using a DTG‐based ART for all PWH [22].

Sex disparities in DTG uptake follow a sadly familiar pattern of disparate treatment based on sex and gender among PWH. An American study found in 2005 that women were less likely to receive ART, pneumocystis prophylaxis and hepatitis C screening when compared to men [23]. A study from the TREAT Asia HIV Observational Database (TAHOD), a collaborative observational cohort study involving 21 sites in 12 countries in the Asia‐Pacific region, found in 2015 significant sex differences in the choice of initial ART, with more women receiving NNRTI‐based ART and a higher likelihood of women to change the ART due to toxicity and side effects [24]. Despite efavirenz‐based ART recommended as first‐line for over 15 years, a global study noted a lower proportion of women received efavirenz compared to men across all regions from 2003 to 2014 [25, 26]. Foreshadowing the recent sex disparities in DTG use, the sex disparities of efavirenz access found might reflect the historical concerns regarding the potential teratogenicity of efavirenz [27, 28, 29, 30]. Our study is consistent with an International epidemiology Databases to Evaluate AIDS (IeDEA) analysis evaluating the disparities in DTG uptake affecting women of reproductive age in 11 low‐ and middle‐income countries in Asia, Latin America and the sub‐Saharan region, from January 2017 through March 2020 [31]. This study found a lower risk of access to DTG among women aged 16–49 years compared to men in the same age group, while they found no difference between both sexes in PWH aged 50 and over. Our study adds to this data by demonstrating the lasting effects of the DTG warning on sex disparities even in more recent years, estimating the difference among ART‐naïve and ART‐experienced PWH.

Our study suggests that the DTG uptake was very heterogeneous across CCASAnet sites. As the first country in the LAC region and one of the first countries in the world to implement DTG as first‐line HIV treatment, only sites in Brazil contributed data in the period prior to the WHO warning [14]. In the period during the DTG warning, only sites in Brazil and Haiti contributed data. Chile and Honduras provided large‐scale DTG only after WHO updated recommendations in July 2019, sites in Peru and Argentina were not included as their general rollout of DTG was even later, and site Mexico did not use DTG as the preferred regimen. This heterogeneity may have biased/impacted our results, though we attempted to minimize this bias by adjusting and stratifying regression models for site. The implementation of DTG in sites in Haiti and Brazil appeared more widespread and uniform than in sites in Chile and Honduras, which could be related to differences in HIV public policies and public health systems. In Brazil, all ART is distributed exclusively through the public health system and the prescription of ART must follow national protocols [5]. Haiti was one of the countries to rapidly transition to DTG as a result of a national recommendation in November 2018, a swift national plan, international cooperation and drug availability in the country [32, 33]. HIV treatment is provided by both the public and private health sectors in Chile and Honduras, and both are still expanding universal access to HIV treatment [34, 35]. In Chile, although ART regimens containing integrase inhibitors have been recommended in the country since 2017, the adoption of DTG as the first line was still at an early stage [36]. As DTG rollout was not homogenous throughout LAC, its uptake also likely influenced our analysis.

Among ART‐experienced PWH in our study, individuals on their first ART regimen were more likely to start DTG. Most studies favouring the switch to DTG compared this drug with efavirenz, demonstrating its superiority [3, 4, 18, 37, 38, 39]. The sensitivity analysis performed among ART‐experienced PWH, disaggregating Haiti from the other countries, revealed that outside Haiti, not being on the first ART regimen and having a detectable viral load (VL) increased the probability of switching to DTG, which may suggest the role of DTG as rescue therapy in PWH with potential therapeutic failure or poor adherence detected with previous NNRTI‐based regimens [40, 41].

Finally, the history of tuberculosis was a negative predictor of starting DTG, both for ART‐naive and ART‐experienced PWH in our cohorts. Possible reasons were the fear of rapid immunologic recovery and the potential risk of immune reconstitution syndrome in PWH with tuberculosis, as well as concerns regarding the coadministration of DTG with rifampicin despite available data on the efficacy, tolerability and safety of DTG use in advanced HIV and co‐infection with tuberculosis [37, 42].

This study has some important limitations. Differences in the DTG uptake across the countries might have affected the results, with data from the pre‐ and during warning period reflecting what was happening at sites in Brazil and Haiti only. Further, there were significant differences in the proportion of females between the countries. We attempted to examine these potential biases through planned sensitivity analyses stratifying by sites in Haiti and all other countries and results regarding sex disparities were largely consistent. Another limitation of our study is that most countries are represented by only one clinic, located in urban areas and often in tertiary care centres. As such, the results are not representative of the entire country or region. We also had a high proportion of viral load missingness, which limited the study ability to look at viral outcomes after ART start. We attempted to address this issue with sensitivity analysis and multiple imputation. Our cohort had a limited number of transgender individuals identified, and we could not look at disparities involving them. We also lacked information on pregnancies and could not look at differences in DTG use specifically related to pregnancies to explain sex differences.

In this large cohort study including sites from LAC, we observed persistent sex disparities in DTG initiation among PWH. This disparity was greatest among young ART‐experienced PWH and was persistent despite updated international guidance recommending DTG for all PWH. During the DTG warning, mathematical models signalled that, even in a scenario of a potential increase in NTD risk, the use of a DTG‐based regimen would result in lower mortality among women and decreased risk of HIV transmission [43]. Women have historically been underrepresented in clinical trials, and pregnant or lactating women are often excluded per protocol due to safety concerns. In addition, the potential for pregnancy influences women's access to certain medications, as seen in the context of ART selection [44]. The emergency modification of HIV health policies due to concerns over a rare and potential teratogenic effect—which was later not confirmed—highlighted the urgent need to advance towards gender‐ and sex‐equitable treatment and care. To avoid disparities, the perspectives and needs of women living with HIV must be central to studies focused on their health, as well as in the development of recommendations and health policies that impact them. Policymakers should monitor DTG uptake to ensure that disparities identified in various studies are addressed through continued education for communities and healthcare professionals and by disseminating information through active organizations advocating for women living with HIV.

## 5 CONCLUSIONS

Younger females with HIV in LAC continue to experience disparities in access to DTG‐containing ART, despite the update of the WHO recommendation reinforcing the importance of women's access to the best ART available, at any time of their life cycle. It is urgent to mobilize researchers, clinicians and civil society to guarantee equity in ART and to repair the consequences of the DTG warning. Future decisions regarding HIV care and ART must include women living with HIV in the decision‐making process so that we can not only learn from the past but mobilize to reduce persistent disparities. It is crucial to integrate sex and gender‐based analyses to advance gender‐ and sex‐equitable treatment and care.

These findings on the continued sex disparities suggest that the same importance given to communicating potential teratogenic effects, which have historically impacted women's health, should be given to communicating the reversal of these alerts and updates on scientific evidence. Communication from large organizations, as the WHO did at the time of the alert, as well as from other international and national regulatory agencies, must reach health professionals, managers and the community in general, and potentially impacted women more specifically, with the same speed and emphasis. The same light should also be shone on important continuing education forums such as congresses and national and international scientific journals, to communicate updates on the initial information more accurately and forcefully and mitigate the impact on women's health.

## COMPETING INTERESTS

All authors declare no conflicts beyond the funding listed above.

## AUTHORS’ CONTRIBUTIONS

FFF, BES, JLC and EMJ conceived and designed this analysis. EMJ, JLC and BES supervised the analysis and the manuscript preparation. FFF reviewed the literature, interpreted the findings and drafted the manuscript. PR and BES performed the statistical analyses. BG, SWC and VGV were involved in revising the manuscript for important intellectual content. FGFF, MFR, DMM, VR, DV and FM critically reviewed it. All authors read and approved the final version.

## FUNDING

This work was supported by the NIH‐funded Caribbean, Central and South America network for HIV epidemiology (CCASAnet), a member cohort of the International Epidemiologic Databases to Evaluate AIDS (leDEA) (U01AI069923), specific funding provided by the Fogarty International Center as part of the Fogarty IeDEA Mentorship Program. This award is funded by the following institutes: the National Institute of Allergy and Infectious Diseases (NIAID), the *Eunice Kennedy Shriver* National Institute of Child Health and Human Development (NICHD), the National Cancer Institute (NCI), the National Institute of Mental Health (NIMH), the National Institute on Drug Abuse (NIDA), the National Heart, Lung, and Blood Institute (NHLBI), the National Institute on Alcohol Abuse and Alcoholism (NIAAA), the National Institute of Diabetes and Digestive and Kidney Diseases (NIDDK), the Fogarty International Center (FIC) and the National Library of Medicine (NLM).

Dr. Grinsztejn work was also supported by FAPERJ Grant, Number E‐26/200.946/2022 (268813), and CNPQ Grant, Number 313265/2023‐2.

## DISCLAIMER

The content is solely the responsibility of the authors and does not necessarily represent the official views of the National Institutes of Health.

## Supporting information




**Figure S1**. Proportion of dolutegravir initiation among treatment naïve and treatment experienced people with HIV, over calendar time, by site and by sex.

**Table S1**. Adjusted prevalence risk ratios of starting dolutegravir among ART‐naïve with HIV in clinical sites in Haiti and all other sites, by sex, period, and age.
**Table S2**. Adjusted hazard ratios of starting dolutegravir among ART‐experienced with HIV in clinical sites in Haiti and all other sites, by sex, period, and age.
**Table S3**. Characteristics of ART naïve PWH with al least 12 months follow up after the date of dolutegravir availability in the country, and with at least one known HIV viral load result in that period, from 2017 to 2022.
**Table S4**. Adjusted prevalence risk ratio of achieving HIV RNA <50 copies/mL within first year of ART among ART‐naive people with HIV, excluding DTG use.
**Table S5**. Adjusted prevalence risk ratio of achieving HIV RNA <50 copies/mL within first year of ART among ART‐naive people with HIV, including no HIV RNA available as failure outcome.

## Data Availability

All analyses were conducted in R version 3.4.3. The analysis code is posted at https://github.com/shepheb1/ArchivedAnalyses/tree/main/Fonseca‐et‐al‐DTG‐2025
