# Supplementary material for: Persistent sex disparities in access to dolutegravir‐based antiretroviral therapy in Latin America and the Caribbean: results from a retrospective observational study using data from 2017 to 2022
Source: J Int AIDS Soc. 2025 Jul 9;28(7):e26470. doi: 10.1002/jia2.26470 (PMC12241694; doi:10.1002/jia2.26470)
Supplement: Supplementary file 1 — Figure S1. Proportion of dolutegravir initiation among treatment naïve and treatment experienced people with HIV, over calendar time, by site and by sex. [file JIA2-28-e26470-s002.docx]

**Supplemental Figure 1 - Proportion of dolutegravir initiation among treatment naïve and treatment experienced people with HIV, over calendar time, by site and by sex.**


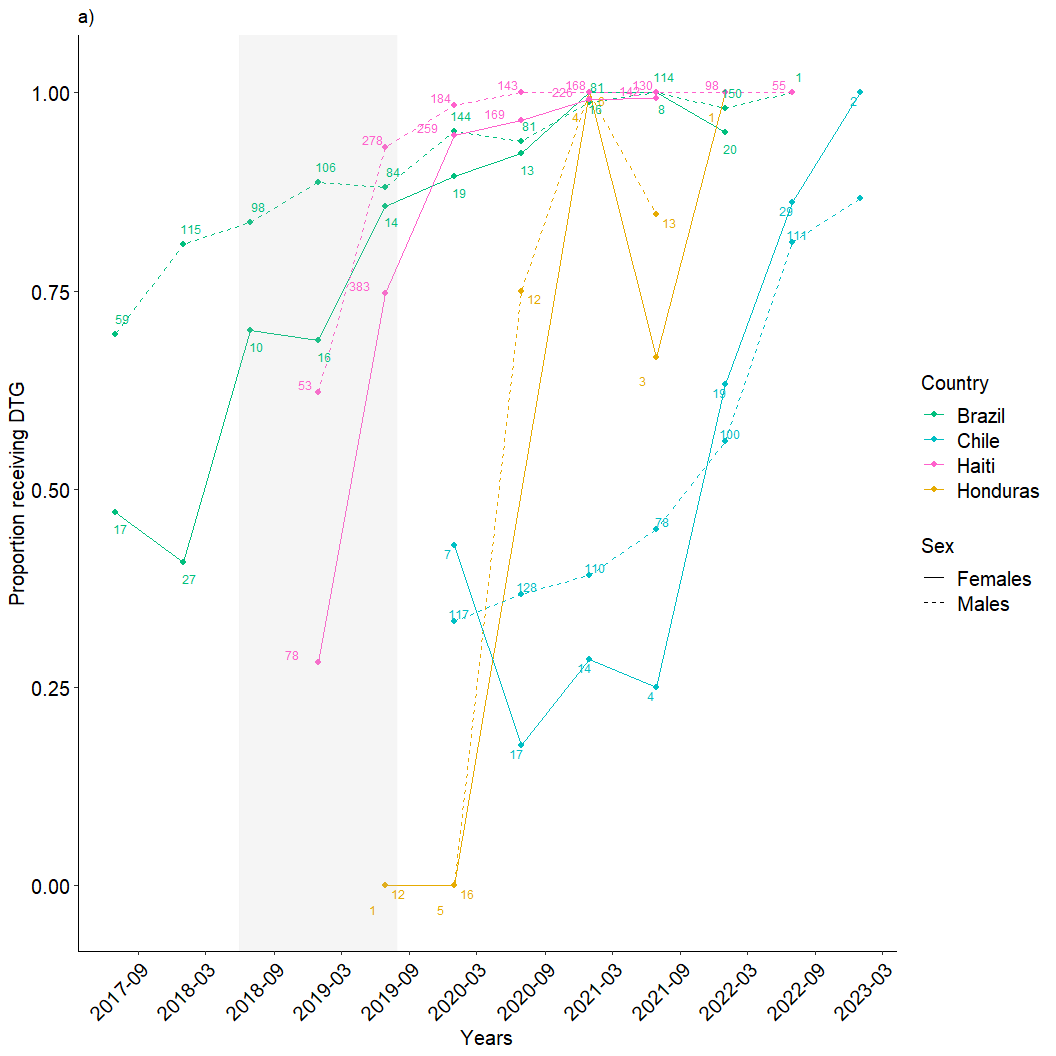


**A**


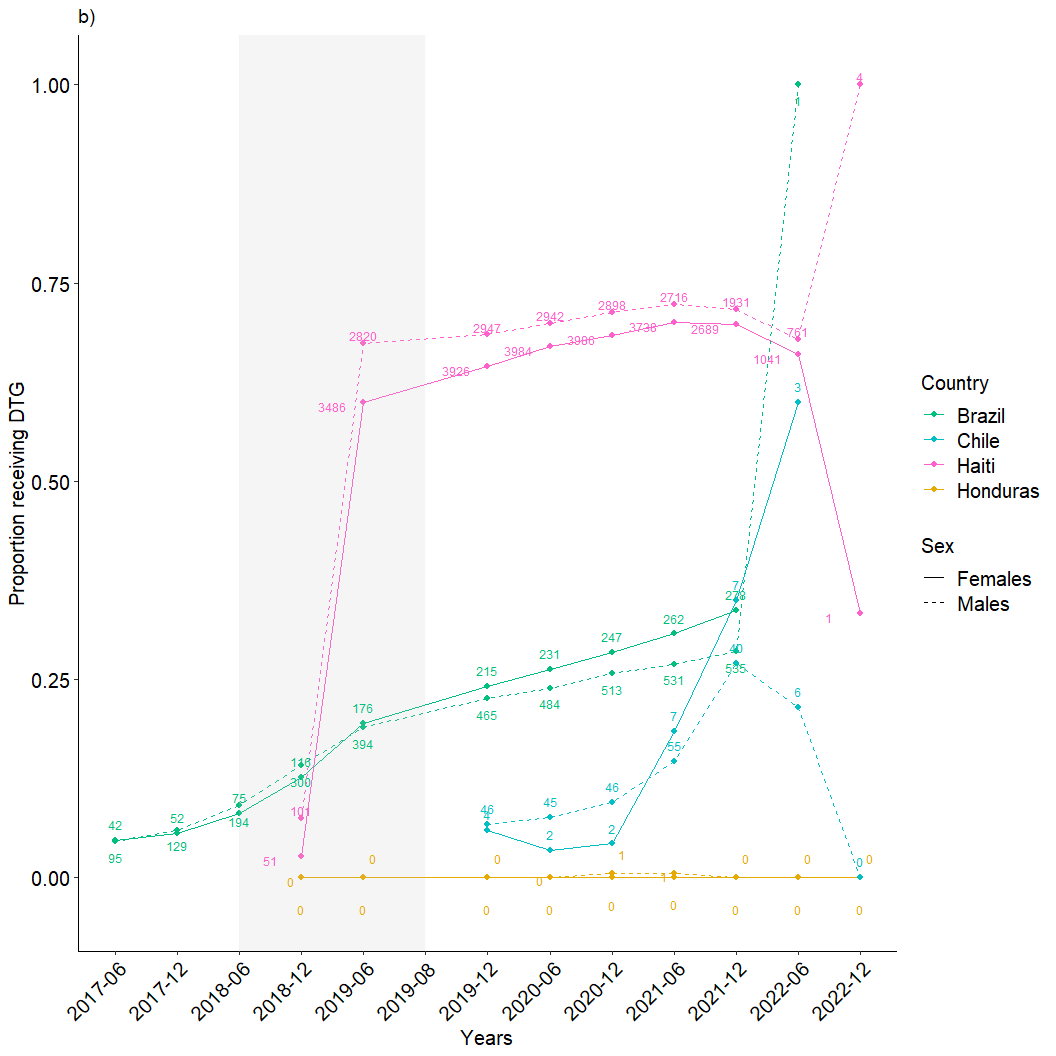


**B**

**Note**: **A.** Treatment naïve people with HIV. **B.** Treatment experienced people with HIV.

Darker area in the graph represents the dolutegravir (DTG) alert period (from May 2018 when the World Health Organization cautioned about the DTG use during the periconceptional period because of a potential teratogenic effect possible related to DTG. That period was considered to last until the WHO updated antiretroviral and HIV care and treatment guidelines on July 2019, reinforcing the role of DTG as the preferred antiretroviral to compose the first line and second-line treatment for all populations, including pregnant women and those of childbearing potential. The numbers on the lines represents the total number of people with HIV on ART in the semester.
