# Supplementary material for: Persistent sex disparities in access to dolutegravir‐based antiretroviral therapy in Latin America and the Caribbean: results from a retrospective observational study using data from 2017 to 2022
Source: J Int AIDS Soc. 2025 Jul 9;28(7):e26470. doi: 10.1002/jia2.26470 (PMC12241694; doi:10.1002/jia2.26470)
Supplement: Supplementary file 2 — Table S1. Adjusted prevalence risk ratios of starting dolutegravir among ART‐naïve with HIV in clinical sites in Haiti and all other sites, by sex, period, and age. Table S2. Adjusted hazard ratios of starting dolutegravir among ART‐experienced with HIV in clinical sites in Haiti and all other sites, by sex, period, and age. Table S3. Characteristics of ART naïve PWH with al least 12 months follow up after the date of dolutegravir availability in the country, and with at least one known HIV viral load result in that period, from 2017 to 2022. Table S4. Adjusted prevalence risk ratio of achieving HIV RNA <50 copies/mL within first year of ART among ART‐naive people with HIV, excluding DTG use. Table S5. Adjusted prevalence risk ratio of achieving HIV RNA <50 copies/mL within first year of ART among ART‐naive people with HIV, including no HIV RNA available as failure outcome. [file JIA2-28-e26470-s001.docx]

**Supplemental Table 1. Adjusted prevalence risk ratios of starting dolutegravir among ART-naïve with HIV in clinical sites in Haiti and all other sites, by sex, period, and age.**

| **COMPARISON** | | **PWH in Haiti site** | | **PWH in all other countries** | | |
| --- | --- | --- | --- | --- | --- | --- |
|  |  | **aPR (95%CI)†** | **p-value** | **aPR (95%CI)†** | **p-value** |  |
| **SEX** | 16-49 years pre/during-warning: females vs. males | 0.75 (0.70-0.81 | <0.001 | 0.77 (0.64-0.93) | <0.001 |  |
|  | ≥50 years pre/during warning: females vs. males | 1.05 (0.94-1.17) | 0.39 | 0.85 (0.58-1.23) | 0.39 |  |
|  | 16-49 years post-warning:  females vs. males | 0.98 (0.97-0.99) | <0.001 | 1.00 (0.91-1.11) | 0.93 |  |
|  | ≥50 years post-warning:  females vs. males | 1.01 (0.99-1.02) | 0.25 | 1.09 (0.88-1.35) | 0.41 |  |
| **PERIOD** | Females 16-49 years:  pre/during vs. post-warning | 0.69 (0.65-0.74) | <0.001 | 0.68 (0.55-0.83) | <0.001 |  |
|  | Males 16-49 years:  pre/during vs. post-warning | 0.90 (0.87-0.93) | <0.001 | 0.89 (0.85-0.92) | <0.001 |  |
|  | Females ≥50 years:  pre/during vs. post-warning | 0.92 (0.86-0.98) | 0.009 | 0.55 (0.38-0.79) | 0.001 |  |
|  | Males ≥50 years:  pre/during vs. post-warning | 0.88 (0.81-0.97) | 0.007 | 0.71 (0.56-0.89) | 0.003 |  |
| **AGE** | Females pre/during-warning:  16-49 vs. ≥50 years | 0.74 (0.67-0.81) | <0.001 | 1.05 (0.73-1.53) | 0.78 |  |
|  | Females post-warning:  16-49 vs. ≥50 years | 0.98 (0.97-0.99) | <0.001 | 0.85 (0.70-1.04) | 0.11 |  |
|  | Males pre/during-warning:  16-49 vs. ≥50 years | 1.03(0.93-1.13) | 0.58 | 1.16(0.96-1.40) | 0.12 |  |
|  | Males post-warning:  16-49 vs. ≥50 years | 1.01 (0.99-1.01) | 0.24 | 0.93 (0.81-1.06) | 0.29 |  |

**Notes:** 
† Multivariable modified Poisson regression model including sex-period-age interaction term, site (for model with PWH from all other countries), and tuberculosis at baseline.

Abbreviations used:

95%CI: 95% confidence interval

aPR: adjusted prevalence risk ratio

ART: antiretroviral

DTG: dolutegravir

Vs.: *versus*

aHR: adjusted hazard ratio

CI: confidence interval

**Supplemental Table 2. Adjusted hazard ratios of starting dolutegravir among ART-experienced with HIV in clinical sites in Haiti and all other sites, by sex, period, and age.**

| **COMPARISON** | | **PWH in Haiti site** | | **PWH in all other countries** | | |
| --- | --- | --- | --- | --- | --- | --- |
|  |  | **aHR (95%CI)†** | **p-value** | **aHR (95%CI)‡** | **p-value** |  |
| **SEX** | 16-49 years pre/during-warning: females vs. males | 0.65 (0.62-0.69) | <0.001 | 0.86 (0.68-1.08) | 0.19 |  |
|  | ≥50 years pre/during warning: females vs. males | 1.07 (0.99-1.15) | 0.09 | 0.94 (0.73-1.22) | 0.65 |  |
|  | 16-49 years post-warning:  females vs. males | 0.55 (0.48-0.64) | <0.001 | 1.27 (0.96-1.68) | 0.09 |  |
|  | ≥50 years post-warning:  females vs. males | 1.22 (0.97 – 1.53) | 0.09 | 1.27 (0.92-1.76) | 0.14 |  |
| **PERIOD** | Females 16-49 years:  pre/during vs. post-warning | 1.03 (0.92-1.15) | 0.64 | 1.07 (0.66-1.75) | 0.79 |  |
|  | Males 16-49 years:  pre/during vs. post-warning | 0.87 (0.77-0.99) | 0.038 | 1.59 (1.02-2.47) | 0.039 |  |
|  | Females ≥50 years:  pre/during vs. post-warning | 1.07(0.91-1.27) | 0.410 | 0.84(0.30-2.39) | 0.75 |  |
|  | Males ≥50 years:  pre/during vs. post-warning | 1.23(1.03-1.47) | 0.022 | 1.14(0.79-1.66) | 0.49 |  |
| **AGE** | Females pre/during-warning:  16-49 vs. ≥50 years | 0.59 (0.55-0.62) | <0.001 | 0.60 (0.45-0.80) | <0.001 |  |
|  | Females post-warning:  16-49 vs. ≥50 years | 0.61 (0.51-0.74) | <0.001 | 0.47 (0.33-0.67) | <0.001 |  |
|  | Males pre/during-warning:  16-49 vs. ≥50 years | 0.96 (0.90-1.03) | 0.23 | 0.66 (0.54-0.80) | <0.001 |  |
|  | Males post-warning:  16-49 vs. ≥50 years | 1.35 (0.61-0.90) | 0.003 | 0.47 (1.66-2.70) | <0.001 |  |

**Notes:** 
† Multivariable Cox proportional hazard model, including sex-period-age interaction term and additional covariates of tuberculosis at baseline, number of previous ART regimens, HIV RNA at baseline, and stratified by site. Missing variables were multiply imputed.

Abbreviations used:

95%CI: 95% confidence interval

aPR: adjusted prevalence risk ratio

ART: antiretroviral

DTG: dolutegravir

Vs.: *versus*

aHR: adjusted hazard ratio

CI: confidence interval

**Supplemental Table 3. Characteristics of ART naïve PWH with al least 12 months follow up after the date of dolutegravir availability in the country, and with at least one known HIV viral load result in that period, from 2017 to 2022.**

|  | **HIV viral load category** | |
| --- | --- | --- |
|  | **Undetectable**  N= 2443 | **Detectable**  N = 304 |
| **Age¹, median (IQR)** | 35 (28-43) | 32 (26-40) |
| **Age group², n (%)** |  |  |
| 16-49 years | 2106 (88) | 277 (12) |
| > 50 years | 337 (93) | 27 (8) |
| **Sex², n (%)** |  |  |
| Female | 963 (88) | 131 (12) |
| Male | 1480 (90) | 173 (10) |
| **Country site², n (%)** |  |  |
| Brazil | 653 (91) | 64 (9) |
| Chile | 254 (82) | 54 (18) |
| Haiti | 1499 (89) | 184 (11) |
| Honduras | 37 (95) | 2 (5) |
| **Time period**²**, n (%)**† |  |  |
| Pre-warning | 227 (89) | 27 (11) |
| During warning | 810 (88) | 113 (12) |
| Post-warning | 1406 (90) | 164 (10) |
| **HIV RNA (log_10_), median (IQR)** † | 4.6 (3.9-5.2) | 5.3 (4.6-5.8) |
| Missing | 1539 | 186 |
| **CD4 cell count** **(cells/mm³), median (IQR)** † | 343 (163-557) | 209 (83-388) |
| **CD4 cell count**² **(cells/mm³), n (%)**† |  |  |
| <350 | 801 (87) | 119 (13) |
| >350 | 773 (94) | 53 (7) |
| Missing | 869 | 132 |
| **TB history², n (%)**† | 93 (82) | 21 (18) |
| **AIDS defining illness (except TB) ², n (%)**† | 125 (78) | 36 (22) |
| **ART regimen**²**, n (%)**† |  |  |
| DTG-based | 2068 (91) | 214 (9) |
| INSTI-other based‡ | 158 (83) | 32 (17) |
| NNRTI-based | 167 (79) | 45 (21) |
| PI-based | 43 (77) | 13 (23) |
| Other § | 7 (100) | 0 (0) |

**Notes:**ART: antiretroviral, DTG: dolutegravir, IINSTI: integrase inhibitor, IQR: interquartile range, PI: protease inhibitor, NNRTI: non-nucleotide reverse transcriptase inhibitor, NA: not applicable

PWH: person living with HIV, TB: tuberculosis, VL: viral load. † Baseline time point refers to ART initiation among treatment-naïve PHIV. ‡ INSTI-other: not DTG based, example raltegravir or bictegravir. § Other ART: fusion inhibitors (enfuvirtide) and R5 receptor antagonists (maraviroc). 1 median (IQR), 2 n (%)

**Supplemental** **Table 4. Adjusted prevalence risk ratio of achieving HIV RNA <50 copies/mL within first year of ART among ART-naive people with HIV, excluding DTG use**

|  | | **aPR (95%CI)†** | **p-value** |
| --- | --- | --- | --- |
| **SEX** | 16-49 years post-warning:  females vs. males | 1.03 (0.96-1.10) | 0.43 |
| **PERIOD** | Males 16-49 years:  pre/during vs. post-warning | 1.01 (0.94-1.08) | 0.81 |
| **AGE** | Males post-warning:  ≥50 years vs. 16-49 years | 0.99 (0.95-1.03) | 0.62 |

† Multivariable modified Poisson regression model including sex-period-age interaction term and additional covariates of site and tuberculosis at baseline.

Abbreviations used:

95%CI: 95% confidence interval

aPR: adjusted prevalence risk ratio

ART: antiretroviral

DTG: dolutegravir

Vs.: *versus*

CI: confidence interval

**Supplemental Table 5. Adjusted prevalence risk ratio of achieving HIV RNA <50 copies/mL within first year of ART among ART-naive people with HIV, including no HIV RNA available as failure outcome**

|  | | **aPR (95%CI)†** | **p-value** |
| --- | --- | --- | --- |
|  | **DTG vs. no DTG** | 1.19 (1.10-1.30) | <0.001 |
| **SEX** | 16-49 years post-warning:  females vs. males | 1.02 (0.90-1.15) | 0.78 |
| **PERIOD** | Males 16-49 years:  pre/during vs. post-warning | 1.05 (0.94-1.18) | 0.36 |
| **AGE** | Males post-warning:  ≥50 years vs. 16-49 years | 0.92 (0.86-1.10) | 0.66 |

† Multivariable modified Poisson regression model including sex-period-age interaction term and additional covariates of site and tuberculosis at baseline.

Abbreviations used:

95%CI: 95% confidence interval

aPR: adjusted prevalence risk ratio

ART: antiretroviral

DTG: dolutegravir

Vs.: *versus*

CI: confidence interval
